# Effects of Reflective Labyrinth Walking Assessed Using a Questionnaire

**DOI:** 10.3390/medicines5040111

**Published:** 2018-10-17

**Authors:** Daniele S. Lizier, Reginaldo Silva-Filho, Juliane Umada, Romualdo Melo, Afonso Carlos Neves

**Affiliations:** 1 Academia Brasileira de Estudos em Medicina Chinesa, São Paulo 03045-002, Brazil; regis@ebramec.com.br (R.S.-F.); judaniee@gmail.com (J.U.); romualdomelo@gmail.com (R.M.); afonsocnn@gmail.com (A.C.N.); 2 Faculdade Brasileira de Medicina Chinesa, São Paulo 03045-002, Brazil

**Keywords:** meditation, labyrinth, mind, walking meditation

## Abstract

**Background:** Meditation as it is currently known is an ancient practice, which can be traced back to Asian traditions. With the proper technique, a state of physical relaxation and respiratory balance can be reached naturally and spontaneously. This paper considers meditative labyrinth walking to be a unique expression of Dr. Lauren Artress’ work, who studied and applied the image of the labyrinth on the floor of the Chartres Cathedral in France. **Methods:** This study used a qualitative approach. It is a cross-sectional non-randomized study, conducted at an institute for psychotherapies with a sample of 30 participants. **Results:** 99% of the group reported feeling emotional distress caused by the feeling of a longer walk on the way out, 21% reported feeling the same while walking the path, and 41% at the beginning. The remaining participants felt lost in time and space. **Conclusions:** This study showed that the practice of labyrinth walking is a physical, emotional, and sensory experience. On the clinical level, correlating this experience to the planning of care seems to be particularly relevant.

## 1. Introduction

### 1.1. Meditation

The Brazilian Portuguese word for “meditation” is “meditação”. In Portuguese, it may have several meanings. The Brazilian Portuguese monolingual dictionary Aurélio defines “meditação” as a feminine noun derived from the verb “meditar” (to meditate), to consider, to think about, to project, to intend, to reflect [1].

Meditation as it is currently known is an ancient practice, which can be traced back to Asian traditions. It is particularly related to the philosophies of Yoga and Buddhism. With the proper technique, a state of physical relaxation and respiratory balance can be reached naturally and spontaneously [2]. However, the term is also used to refer to practices fostered by certain religions, including Christianity, Judaism, Islam, Taoism, and Shamanism involving shifting consciousness from the outer world to the inner world [3]. Another recent form of meditation is mindfulness [4]. 

The National Institute of Health (NIH), a US agency regulating medical research, has formally acknowledged meditation as a therapeutic practice that may be associated with conventional medicine [5].

The Brazilian Ministry of Health has included meditation in its National Policy on Integrative and Complementary Practices (PNPIC), enacted 3 May 2006, as per Ordinance 971/GM/MS published in Edition 84 of the Official Government Gazette on 4 May 2006, Section 1, page 20. This policy encourages Brazilian health centers and public hospitals to offer meditation as a therapeutic alternative. These government actions are signs of a tendency to face meditation not only for the purposes of mental and spiritual well-being, but also for physical well-being [6]. Behavioral and neurophysiological studies show that meditation not only enhances attention, but also increase physical and psychological responses [7,8].

In terms of scientific research, records show that the potentialities of meditation have been discussed as far back as 1936, but it was only in the 60’s that meditation became the object of strict research [9].

Currently, studies suggest that meditation may physically change both the brain and the body, aiding in the improvement of a host of physical and psychic conditions. In a study conducted in 2012, researchers compared brain images of 50 adult meditation practitioners and non-practitioners, each. Results suggest that longer lasting meditation practitioners presented more folding (gyri) of the outer layer of the brain. This process known as gyrification is believed to increase the capacity of the brain for processing information [10]. A 2013 literature review of three studies suggests that meditation may delay, halt, or even reverse normal age-related changes in the brain [11].

Studies suggest that research conducted over the last few decades largely supports the claim that conscious meditation (mindfulness), if regularly practiced, may reduce stress, promote health and have beneficial effects on physical and mental health and cognitive performance. Few Brazilian institutions are currently at the forefront of mindfulness research, training, and treatment. 

Recent neuroimaging studies have begun correlating areas of the brain and networks related the aforementioned positive effects [12].

A study, showed that meditation training improves brain efficiency for attention and impulse control. This study [13,14] compared a group of 20 regular meditators with a group of 19 non-meditators during an functional magnetic resonance imaging-adapted Stroop Word-Color Task (SWCT). The Stroop Task is widely used in neuropsychology. The task allows for the detection of neurological and brain conditions and may be used to assess attention and monitor cognitive dysfunction. It assesses selective attention, the capacity to focus on an activity and inhibit the propensity to respond impulsively, in addition to the speed of information processing [14]. The task involves circuits subserving attention, working memory, response selection and inhibition, motor planning, and motor response among others [15]. Regular meditation practitioners showed an activation of less regions of the brain in comparison to non-meditators during the performance of an attention task. This suggests that meditation training may improve brain efficiency for attention and impulse control [15]. 

In 2016, a study presented an overview of studies conducted in his laboratory on neural changes associated with various forms of meditation. Distinctions among three major forms of meditation practice were made: focused attention, open monitoring, and positive affect training [16]. Each of these forms of meditation has different neural and behavioral effects. From the perspective of Western neuroscience, different forms of meditation can be conceptualized as mental training to promote the regulation of emotion and attention, also reviewed some longitudinal studies that tracked changes over time with meditation practice. In addition to the neural changes that have been observed, he summarized changes that have been found in peripheral biology that may modulate physical health and illness. The central brain circuitry of emotion is especially implicated in peripheral biological changes that have consequences for health. The overall conclusions from these studies are that one can transform the mind through meditation and thereby alter the brain and the periphery in ways that may be beneficial for mental and physical health, and for well-being [16].

Today, meditation is the subject of academic studies, it is employed in the corporate world and it is discussed by the media

A study [17] characterizes meditation as a procedure containing the following operation parameters: 

(1) Use of a specific technique (clearly defined).

(2) Muscle relaxation at some moment of process.

(3) Logic relaxation, which is basically the act of not rationalizing the possible psycho-physical effects, including not incurring in any analyzing nor judging during the process of meditation.

(4) Reaching a self-induced state, which refers to the ability of self-applying a therapeutic method previously taught by an instructor (i.e., meditation must be perfectly feasible to be done at home).

(5) Ability to self-focus (anchor), i.e., in order to begin meditating, the individual would need clear anchors that would allow them to monitor focus. In this phase, an anchor must be actively exercised. Being focused consists of remaining subtly vigilant to the possibility of getting pulled into a train of thought. The loss of the anchor represents the perception of letting oneself get pulled into the thinking mind. Thus, the active exercise of the anchor is the exercise of one’s perception of getting pulled into a sequence of thoughts [17].

Meditation may generate a series of physical and psychological responses aiding in the prevention of several conditions (especially those resulting from the deleterious effects of stress), management of known health conditions, and promoting mental health [15].

All techniques will be effective if the meditator follows the proper operational protocols, even though some techniques may be more popular than others due to having been studied in further detail, such as transcendental meditation, Zazen, Vipassana, and mindfulness. Cardoso claims that techniques with objective anchors are more beneficial to the beginner meditator, who should keep to those techniques until they have exercised meditation to a considerable degree. From that moment on, subjective anchors might be a better fit [17,18].

Meditation may be used for therapeutic purposes if the particulars of the condition under treatment are observed. Not all forms of meditation are necessarily linked to Asian beliefs. For meditation to fulfill its role in complementary and preventive medicine, it must be practice daily and constantly [17].

### 1.2. Meditation and Movement

Walking meditation or meditative walking is a simple and universal practice for improving tranquility, connection, and awareness of the body. It may be practiced regularly, before or after meditating while being still, or at any moment the individual wishes to do so. The art of walking meditation is learning how to sustain awareness while walking. It is using the natural movement of the walk for fostering attention and presence, for being awake in the present moment [19].

Physical activity such as walking impacts mood as it reduces activity in the sympathetic nervous system and the associated hypothalamic-pituitary-adrenal axis responses in the brain [20].

In a study comparing the effects of walking meditation to the effects of a fast-paced walk on individuals with anxiety, meditation was shown to be the preferred method of attenuating the symptoms of anxiety, as compared to a fast-paced walk [21].

Different forms of meditation foster relaxation to variable degrees. This variation may often be attributed to individual factors, rather than necessarily the intrinsic characteristics of meditation—the same applies to meditative labyrinth walking [22,23].

### 1.3. Meditative Labyrinth Walking

This paper considers meditative labyrinth walking to be a unique expression of Dr. Lauren Artress’ (1995) work, who studied and applied the image of the labyrinth on the floor of the Chartres Cathedral in France [24,25].

Lauren is a key catalyst of our emerging awareness of the mind-body connection, the impact of our thoughts on our lives and the importance of living with compassion [23,26]. The labyrinth walking triggers meditation-like processes of Western origins, which can be traced back to penitential practices in the Middle Ages [24,27].

### 1.4. Labyrinths and How to Prepare for Walking Meditation

In this study, bi-dimensional portable labyrinths were painted over circular pieces of duck canvas. Usually, the classic pattern will vary from eleven to seven circuits, depending on size [23,24]. Patterns will lead inwards towards the center, and from the center back out again. Our design did not include any dead-ends. Both the entrance and the exit were unique. All participants were instructed by a facilitator. Participants were to walk a coiled path with a 180-degree turn marking the beginning of a new circuit. They received a brief explanation before beginning a meditative labyrinth walk. They were instructed to remove their shoes, but were allowed the use of shoe covers, if desired [23].

There are three stages to labyrinth walking.

Stage 1: Preparation. Consists of walking the labyrinth towards the center at a rhythm of choice, either slow-or fast-paced.

Stage 2: Illumination. At the center, the individual may choose to sit or stand, assuming the most comfortable position, with eyes either opened or closed (Our design included a mandala; participants could choose to look at it or not).

Stage 3: Restoration. Consists of walking back out away from the center and towards the beginning. Upon exiting the labyrinth, participants were handed two texts for reflection [24].

This activity requires no training nor any kind of extenuating physical movement. Walking the labyrinth is the kind of meditation technique that any person may apply, regardless of prior experience or athletic training. Ultimately, one of the objectives of using the labyrinth is improving attention and judgment-free awareness of the present moment, which is easily reached with the involving (but not frustrating) activity provided by the patterns of the labyrinth. On the other hand, it is also possible to perform a few repetitions with a specific purpose in mind [24].

### 1.5. The Benefits and Effects of Labyrinth Walking 

The labyrinth has been rekindled as a tool for emotional and spiritual support. In communities, churches, schools, parks, clubs, spas, spiritual retreats, and even prisons from all over the world the labyrinth is used as a recreational and anti-stress resource to cope with the troublesome reality of big urban centers [28,29,30].

Hospitals have used labyrinths as a support tool for treating diseases such as cancer. The act of walking a labyrinth awakens the potential for contemplation, reflection, and transformation, according to data from the Oncology Nursing Society. These data indicate that walking a labyrinth is a form of psychoneuroimmunology that may use for integrative patient care. Labyrinths are available to nurses as a tool for aiding patients undergoing oncology treatment to reach a contemplative and altered state of consciousness [30,31,32].

Hospitals are not the only stressful environments benefiting from the practice of labyrinth walking. For example, in Santa Fe, New Mexico, 10 elementary schools built labyrinths on the school grounds, resulting in calmer children and an increase in the ability to focus. Additionally, research from the University of Massachusetts, Amherst, began testing the effects of labyrinth walking on the rate of recidivism among prisoners. Over the course of a six-week study conducted with imprisoned individuals, concluded that walking the labyrinth may positively impact this population’s physical and mental health [31,33].

## 2. Materials and Methods 

This study used the descriptive exposition of the results was made quantitatively and the evaluation by themes was qualitative approach.

The trustworthiness method was synchronous, we observed that the participants classified similar concomitant events in the field of sensory, but it is not a phenomenon of hallucinatory characteristic but of the imaginary.

It is a cross sectional study, conducted at an institute for psychotherapies with a sample of 30 participants.

### Sample and Recruitment

The study participants were 30 members of an integrative therapy institute who volunteered for labyrinth walking. This was a convenience sample of people who have been enrolled in other treatment programs, like for example family therapy, acupuncture and yoga. To recruit volunteers we followed institutional protocol with approval of treatment personnel. The invitation was made by email and Pamphlets explaining briefly the study. An information session was given by one of the researchers to all those interested who wanted to know more about the practice of the project. This was a descriptive pilot study. Participants received specific training on the study protocol by the principal investigator. This met with each participant prior to the activity to obtain written and verbal consent and to administer the questionnaires and the demographic form. All questionnaires were together for ease of completion and confidentiality to high response rate. Responses to the questionnaires were collected after the labyrinth walk activity. The software IBM SPSS Statistics Version 23.0, was used to perform simple statistics (frequencies, sums, and means) for demographic major categories were created based on line by line analysis. All the participants were healthy and did not present any alteration before the participation of the activity.

A questionnaire was drafted to include demographics (sex, age, marital status, level of formal education, use of psychoactive drugs and psychotherapy) and information on the practice of meditation, such as kind of meditation practiced, time, frequency, and duration of practice. The following open question was also included: how is meditation echoed into your life and how do you feel after practicing it? This question allowed for the investigation of participant perception on the effects of practice.

All participants were assessed in a follow up session with a physiotherapist, who checked the filled-out questionnaires. In this study, categories were created for an improved understanding of participant answers and testimonials after the activity. The practical time was 25 min.

## 3. Results

### 3.1. Demographics

Demographic data (sex, age, religion, and level of formal education) showed a majority of female (91%) and a minority of male (9%) participants, whose education level was complete Higher Education (73%) followed by complete Secondary Education (14%), and finally complete Primary Education (13%). In terms of religion, 73% participants were Spiritist, 14% were Buddhist, and 13% were Catholic. Despite most of the population being of the Spiritist belief, answers were analyzed according to categories created by the authors, which were unrelated to any particular belief, as seen on Table 1.

**Figure 1:**
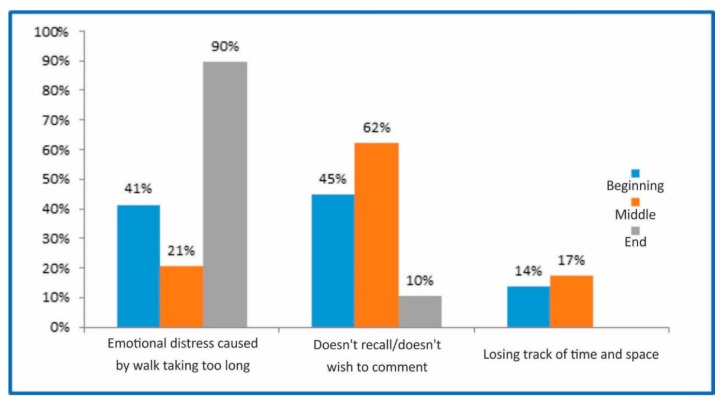
Correlation with time and space: perception and sensation.

Ninety percent of the group reported feeling emotional distress caused by the felling of a longer walk on the way out, 21% reported feeling the same while walking the path, and 41% at the beginning. The remaining participants felt lost in time and space (Figure 1). 

Changes in perception or physical sensation were reported by 86.21% of the group, such as heavy legs or walking on water. The remaining 34.48% reported seemingly hearing sounds that were different from ambient sounds, such as the sound of falling water; 17.24% reported visual perceptions, colors and a seemingly different environment; 13.79% felt the smell of flowers (Table 2).

### 3.2. Recollection of the Walk

**Figure 2:**
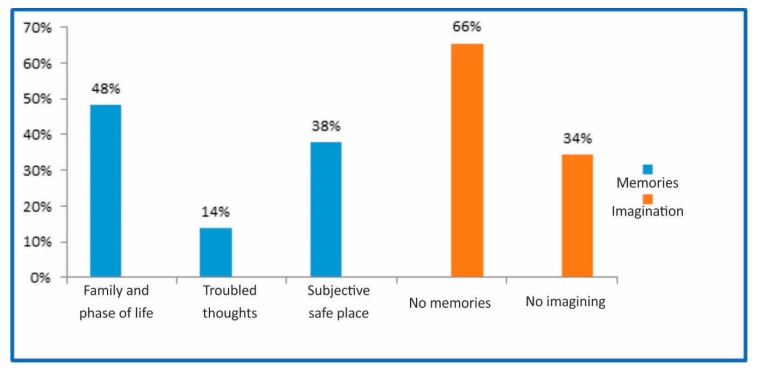
Correlation with memories and the walk.

When participants were questioned about their thoughts during the practice, 66% reported imagine themselves in a safe place known solely by them, 34% reported not having imagined anything, 48% either recalled their family members or a specific phase in life, 14% recalled troublesome matters, and the remaining 38% did not recall anything (Figure 2).

## 4. Discussion

The main goal of this study was assessing the impact of meditative labyrinth walking as practiced by a non-specific group of individuals, including feelings during and after the practice. Curiously, participants identified several themes in connection with this practice, such as bodily perception and sensation, space, time, and memories. The effects of the practice were shown to be individualized. This form of meditation adjusts to both individual personality and present state of mind.

Participant beliefs were included in the questionnaire for the purposes of registering any pre-existing beliefs, either related to religion, spirituality, expectations, or hopes. The World Health Organization (WHO, 1998) defines spirituality as the set of all emotions and convictions of non-tangible nature; the supposition that living entails more than what we fully understand; and questions including the meaning of life, but not limited to any specific belief or religious practice. Thus, belief was included as a cultural factor to be collected along with individual participant data.

Physical and spiritual processes involved in walking the labyrinth were associated to positive clinical outcomes in the literature [32,33].

In modern society, the labyrinth has become a support for personal well-being and self-care. It has become increasingly known as a practice of spiritual care in various settings for health and social care, including cancer care [34,35] and long-term care [36].

### 4.1. Sensory Perception

The purpose of this study was to investigate the effectiveness of a walking meditation in Labyrinth for 25 min on sensory function parameters among a sample of a healthy people group. Changes in perception or physical sensation were reported by 86.21% of the group, such as heavy legs or walking on water. The remaining 34.48% reported seemingly hearing sounds that were different from ambient sounds, such as the sound of falling water; 17.24% reported visual perceptions, such colors and a seemingly different environment; 13.79% felt the smell of flowers. In sum, structural differences resulting from meditation were found in areas that affect awareness, attention, memory, and emotion regulation. At this time, the field of contemplative neuroscience is coming to a consensus about potential applications of mind–body practices, consolidating findings given the wide range in methodologies and research design. To date, the data include structural brain differences, change in neural activity using functional MRI (fMRI), and the type of task used during brain assessment, from resting-state brain to meditation-specific changes (fMRI), and changes in blood flow (fMRI) or positron emission tomography (PET) or single-photon emission computed tomography (SPECT) [37,38].

### 4.2. Body Proprioception

Walking the labyrinth awakens a new form of perceiving the space between the body and the ground, which was reported by participants as changes in proprioception and in the notion of time and space. Changes in perception or physical sensation were reported by 86.21% of the group, such as heavy legs or walking on water.

Another effect may be a new way of organizing time. In a study of the practice of labyrinth walking conducted at the Southwest Centre for Forensic Mental Health Care in St. Thomas, Ontario, participants reported the sensation of escaping daily routine, promoting a change in the hospital environment. Participants noted that labyrinth walking supported the theme of escaping typical daily routine [39]. 

We suggest that the perception of the body and space built while walking an unexpected path activates bodily and spatial memories that may provoke the feeling of existing in a different time reported by walkers of the labyrinth. This may be due to the apprehension of unusual external stimuli [28,40]. Other research suggests that the benefits of mind–body interventions are relayed via the downregulation of stress, mainly through the hypothalamic–pituitary–adrenal (HPA) axis, known for its role in stress control [13,37,38,39,41].

The process of walking into the physical space of the labyrinth and making oneself available to the various surrounding stimuli may provoke a series of effects on the body and the mind that are mediated by the nervous system [42].

### 4.3. Imagination

Sixty-six percent of study participants reported imagining being in a safe place known solely by them. In fact, the labyrinth helps to explore the perception of individual beliefs and to develop an interpretation of that experience with each walk. In 2012, authors [41,42] identified walking the labyrinth as a potential factor for insight development [43]. Additionally, they state each participant’s unique experience is rich and may serve as a basis for individual reflection. States that therapeutic labyrinth walking “can be instrumental in releasing mental and physical tension” [44].

The circle of a labyrinth is a universal symbol for unity and totality that awakens a sense of being connected to other people, to the whole, which innately stimulates memories of our purpose in life [40].

The labyrinths is an archetypal figures that represent paths and journeys of various symbolic meanings. The labyrinth archetype can be found in almost every religion of the world and represents “universal patterns most likely created in the realm of the collective unconscious, birthed through the human psyche and passed down through the ages” [24,40].

### 4.4. Cognitive Function

Many of the participants reported the activation of significant memories and facts during this practice. Notably, meditation has been shown to activate areas of the brain that are implicated in commonly employed tests of cognitive function (e.g., the anterior cingulate cortex and the prefrontal cortex) [37,45]. McMorris’s neuroendocrinological model for exercise-related cognitive benefits [38] suggests that exercise facilitates hypothalamus-induced catecholamine synthesis whereby adrenaline and noradrenaline are released from the adrenal medulla and, subsequently, catecholamines are released in the brain. Norepinephrine and dopamine, for example, are believed to play critical roles in information-processing brain networks. Previous evidence [46,47] also supports the ability of mindfulness meditation to improve aspects of cognitive functioning, such as attention- and memory-related parameters. Similarly, a study of 2007 notes that walking a labyrinth is an opportunity for taking time off the pressure of daily activities [45].

## 5. Conclusions

This study showed that the practice of labyrinth walking is a physical, emotional, and sensory experience. On the clinical level, correlating this experience to the planning of care seems to be particularly relevant.

Further research should be conducted in future studies to verify other experiences that may eventually manifest in different categories.

## 6. Special Considerations

This study showed that the practice of labyrinth walking teaches the patient a new way of addressing their potential for concentration and establishing a connection with their experience at a present moment. Its purpose is to reduce the tendency to and affinity with dysfunctional thoughts. This practice is beneficial and may be used by individuals undergoing therapy or wishing to improve their quality of life.

In the face of abundant positive evidence, future studies of labyrinth-walking-based programs as a form of treatment for a wide range of mental illnesses are warranted.

## Figures and Tables

**Table 1:** Demographics Data.

**Age**	
20–29 years	4%
30–39 years	18%
40–49 years	23%
50–59 years	41%
60–69 years	9%
70–79 years	5%
**Gender**	
Female	91%
Male	9%
**Religion**	
Buddhist	14%
Catholic	13%
Spiritist	73%
**Level of Formal Education**	
Primary Education	13%
Secondary Education	14%
Higher Education	73%

**Table 2:** Perception and Sensation.

Type	%
Olfactory	13.79%
Physical	86.21%
Visual	17.24%
Auditory	34.48%
